# Talking-associated cognitive loads degrade the quality of gaze behavior

**DOI:** 10.1371/journal.pone.0333586

**Published:** 2025-10-06

**Authors:** Takuya Suzuki, Takaji Suzuki, Shintaro Uehara

**Affiliations:** 1 Department of Rehabilitation, Fujita Health University Hospital, Aichi, Japan; 2 Graduate School of Health Sciences, Fujita Health University, Aichi, Japan; 3 Faculty of Rehabilitation, Fujita Health University School of Health Sciences, Aichi, Japan; The Ohio State University, UNITED STATES OF AMERICA

## Abstract

Talking-associated cognitive distractions have been known to significantly impact physical reactions in response to visual information, leading to an increased crash risk while driving. The visuomotor processes required for driving include gaze behavior, cognitive processes, and responsive physical actions. However, how talking-associated cognitive loads affect the quality of gaze behavior remains unclear. Healthy participants performed center-out eye movements toward a peripheral visual target as quickly and accurately as possible under three different conditions: while verbally communicating (talking), listening to audio clips, or performing nothing other than the eye movement task. We found delays in the time needed to react to, move to, and fixate on a peripherally presented visual target in the talking condition compared with the other two conditions. Our results demonstrate that talking-associated cognitive loads are likely to have a strong enough impact to interfere with neural processes for initiating and controlling eye movement. These findings suggest that delayed physical responses and/or impairments in driving performance under cognitively demanding situations may partly result from delayed visual responses to surrounding events, followed by less accurate eye movement control when directing to and maintaining fixation on those stimuli.

## Introduction

Cognitive distraction is known to have a considerable impact on human motor behavior, often leading to interference with tasks that require fine motor skills or precise coordination. This is particularly evident in dual-task scenarios, where individuals perform two tasks simultaneously, such as walking while carrying on a conversation or operating machinery while mentally solving a problem [1–3]. In these situations, cognitive demands can compete for attentional resources, leading to delays, reduced accuracy, or even errors in motor execution ([4] for review).

One of the riskiest of our daily activities that can be disrupted by cognitive distraction is driving a motor vehicle. One conceivable source of distraction is the use of a mobile phone while driving, which results in an increased crash risk that is four times higher than that of normal driving [5]. Impairments in driving performance associated with the manipulation of a mobile phone while driving can be as profound as those associated with driving while drunk [6].

This increased risk appears to be similar for both hand-held and hands-free phones and is unrelated to the content of the conversation. Psychological experiments have shown that talking on the phone while driving, regardless of whether a hands-free function was used, delayed responses to surrounding visual information [6,7], producing comparable crash risks [8]. A systematic review of relevant literature showed that delayed responses to visual stimuli were caused by any of the following conditions: talking with a passenger, talking on a hand-held phone, and talking on a hands-free phone [9,10]. A brain imaging study demonstrated that language comprehension performed while driving draws mental resources away from driving and produces deterioration in driving performance, even when the act of language comprehension does not require holding or dialing a phone [11]. Moreover, delayed responses (e.g., stepping on the brakes) occurred regardless of the content of the conversations, which might suggest a difference in the extent of cognitive demands [12]; however, another work reported that more complex and difficult conversations had greater negative effects on driver distraction [13], as found in a particular impact of cognitive loads on visual attention [14,15]. Altogether, these previous findings suggest that merely being involved in a conversation has the potential to have a substantial impact on the visuomotor processing required while driving.

Visuomotor processes that occur while driving can be divided into several steps: eye movements (e.g., saccades) towards a visual object, object recognition, motor (physical response) planning, and finally, motor execution. Delayed physical responses to external visual events during a conversation are potentially attributed to interference in any of the steps throughout this process. However, it remains unclear whether gaze behavior—essential for obtaining visual information that supplies 90% of the input needed for driving [16]—are subject to interference from talking-associated cognitive loads. Given that driving requires rapid and purposeful gaze shifts (i.e., voluntary saccades) to scan the environment, anticipate hazards, and follow traffic signs etc., interference to the gaze control may have a strong impact on driving quality and thereby safety [17]. Regarding the effects of cognitive loading tasks on visual behavior, previous works have demonstrated that the gaze tended to stay at the center of the field of vision, resulting in fewer shits towards the peripheral visual field [18] and reduced accuracy of gaze landing [19]. Therefore, it is plausible that talking-associated cognitive loads may affect gaze behavior, particularly by delaying responses to peripheral visual targets and reducing the accuracy of subsequent eye movements toward those targets.

To address this question, the present study investigated the effects of talking-associated cognitive loads on the temporal parameters of gaze shifts when responding to peripheral visual targets. Healthy participants performed a center-out eye movement task while verbally communicating, listening to audio clips, or performing nothing other than the eye movement task. For the verbal communication condition, we prepared a set of questions and asked participants to answer them accordingly. We hypothesized that gaze behavior —specifically, the latency to initiate and the time to complete movements toward peripheral targets—would be degraded while talking but not in the other two situations. This assumption is supported by evidence that simply listening to radio clips does not affect visuomotor responses [20,21]. Furthermore, we explored the possibility of direction-specific differences in the effect of talking-associated cognitive loads on gaze behavior.

## Materials and methods

### Participants

The study was reviewed and approved by the Ethics Review Committee of Fujita Health University (approval number: HM18–369 [original] and HM20–073 [revised]) and was conducted in accordance with the principles of the 1964 Declaration of Helsinki, as revised in 2013. A total of 30 participants (mean age, 22.6 years; standard deviation (SD), 4.3; 17 female) were recruited for the study between 11/07/2019 and 13/08/2020, based on the inclusion criterion of having no history of diseases affecting color vision or astigmatism. All the participants provided written informed consent before participating in the study. None of the participants had a history of neurological disease and/or vision disorders, such as color vision defect. The sample size of the present study was determined following psychological experiments exploring the effects of talking-associated cognitive loads on human physical responses (see studies in [7]).

### Center-out eye movement task

**Fig 1:**
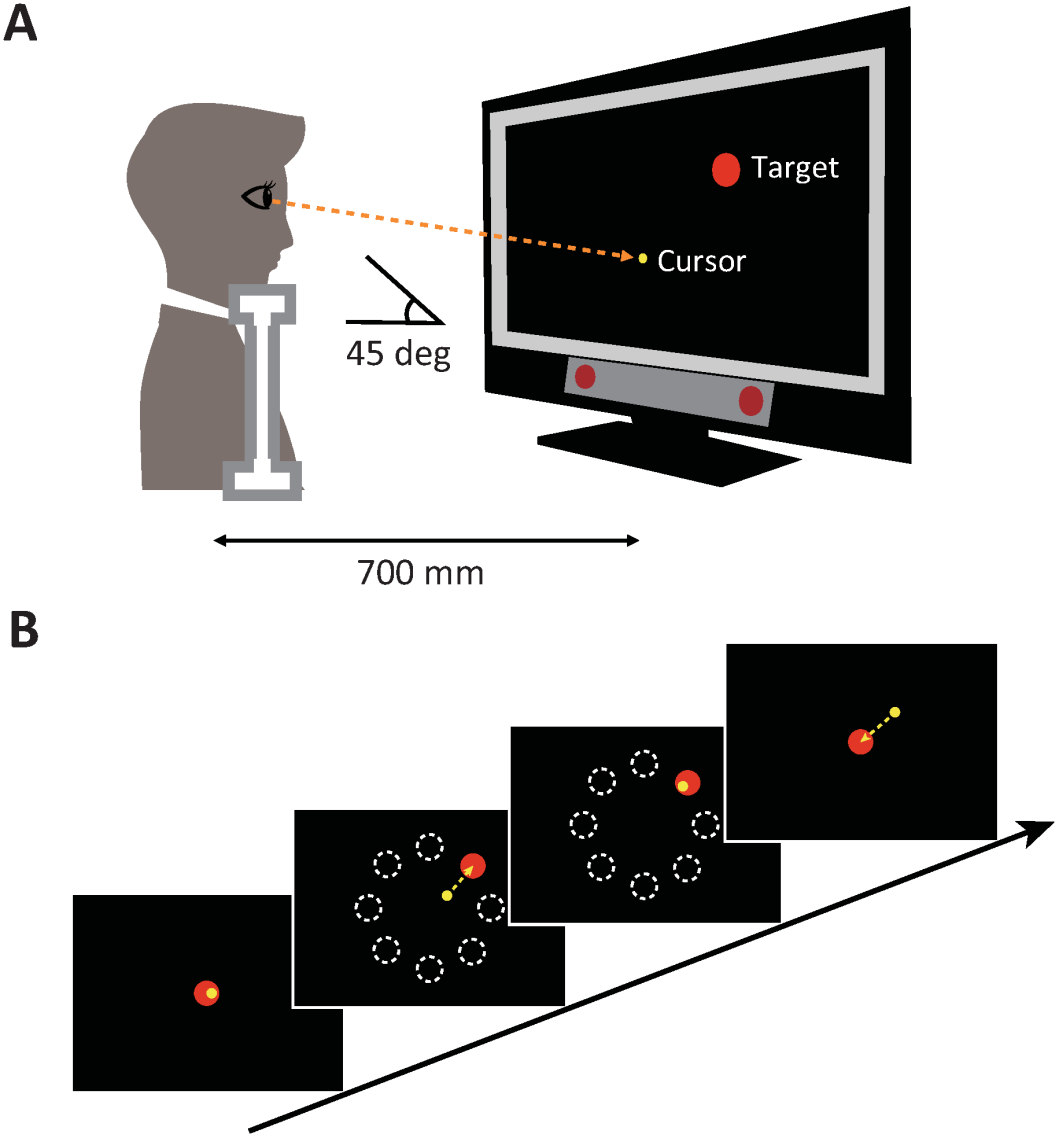
Experimental setup and the eye movement task. A: Experimental setup. Participants repeatedly performed fast and accurate center-out eye movements from the center of the display toward a peripheral visual target. B: A sequence of trials in the eye movement task. After the cursor representing the gaze position had been maintained on the central starting position for 1 s, a target appeared in one of eight locations. Upon presentation of a target, participants started to move their eyes so that the cursor overlapped with the target. After the cursor had been maintained on a target for 1 consecutive second, the target disappeared, and the central starting position reappeared simultaneously.

We asked participants to perform a center-out eye movement task in which they moved their gaze toward a target that appeared at one of eight different locations. Participants sat on a chair with their chin on a chinrest 700 mm in front of a 21-inch computer display (1920 × 1080 pixel resolution, Lenovo, Japan). Their gaze position, tracked by an eye tracker (PCEye Plus, Tobii Dynavox, Sweden) and sampled at 60 Hz through a custom MATLAB program version R2018a (The MathWorks, Inc., Natick, MA, USA), was transformed to the position of a yellow 2-mm-diameter cursor on a black screen. For the calibration of gaze position, participants were instructed to fixate on a series of dots appearing at seven fixed positions on the display, during which their eye movement were recorded using the eye tracker and associated software (Tobii Pro Eye Tracker Manager, Tobii Dynavox, Sweden). Calibration quality was assessed by the software, and recalibration was conducted if necessary to ensure high tracking accuracy. The position of the participant’s eyes and the eye-tracking device was set to 45 ° (Fig 1A). In the task, participants attempted to move the displayed cursor from a red 20-mm-diameter dot centered at the middle of the screen toward a red 20-mm-diameter target displayed in one of eight positions (0, 45, 90, 135, 180, 225, 270, and 315 °) arrayed radially 10 cm from the central starting position (Fig 1B). Participants were instructed to move their gaze toward the target as quickly and accurately as possible.

Each trial started with moving the gaze position such that the cursor was positioned within the starting position. After maintaining this position for 1 s, one of the targets was presented. Upon presentation of the target, participants started to move their eyes so that the cursor overlapped with the target. After the cursor stayed on the target for 1 s, the target disappeared, and the central starting position appeared. Then, participants attempted to move the cursor back to the starting position. The eight different targets were presented pseudo-randomly so that every set of eight consecutive trials included one of each of the target positions.

### Experimental procedure

Participants performed the tasks under three different conditions: 1) verbal communication (talking condition), 2) listening to audio clips (listening condition), and 3) a control condition without additional tasks. For the talking condition, we prepared a specific set of questions and asked participants to answer them accordingly. For this, we selected some items from the Wechsler Adult Intelligence Scale – Third Edition (WAIS-iii), a test most commonly used to investigate intelligence in adults [22] with good validity and reliability [23]. We have selected 14 questions about Information, 10 questions about Vocabulary, and 7 questions about Similarities from one of the subsets of Verbal Comprehension Index. For example, we used some questions that ask participants about specific knowledge such as “Where is the capital of Italy?” We also prepared 14 original questions asking about episodic knowledge, such as “When did you sleep last night?” and “What color shirt did you wear yesterday?” We adopted this setting, i.e., questioning and answering rather than allowing participants to have an open-ended conversation, to limit the range of content in the conversation. In total, 45 possible questions were prepared, and a subset of possible questions were randomly chosen and asked to a participant at a certain timing at an informal pace during performing the task (i.e., one question was not provided per trial). We gave a verbal instruction to the participants that “please try to think deeply and seek an answer based on your experience even if you don’t come up with the right answer. And, if you don’t find the answer, please say so”. During the listening condition, participants were exposed to audio clips from a recitation. The content of the recitation was selected from the famous Japanese novel *I am a cat*. We instructed participants to focus on and understand the content of the novel. In the control condition, no other tasks were provided other than the eye movement task.

Under each experimental condition, participants performed two blocks of 40 task trials (five trials for each of eight directions; 80 trials in total). Participants were allowed to have a 1 min break between the blocks to prevent the effects of fatigue. Each experimental condition was performed on three different days that were separated by at least one day. The order of the three conditions was randomized among participants.

### Data analysis

Regarding the characteristics of gaze behavior, we analyzed reaction, movement, and adjusting times. The reaction time was the duration from when the visual target appeared to when the cursor left the central starting position. The movement time was the duration from when the cursor left the starting position to when the cursor hit the target for the first time. For the adjusting time, we measured the duration from when the cursor hit the target to when the cursor stayed inside the target for 1 s. For each movement parameter, we computed the median of 10 trials for each of the eight directions as a representative proxy for each participant.

### Statistical analysis

To investigate the effects of three experimental conditions on gaze behavior, we applied a repeated-measures analysis of variance (ANOVA_RM_) with within-participant factors for the conditions (talking, listening, and control) and directions (eight directions) for each movement parameter. ANOVA_RM_ was tested for the assumption of homogeneity of variance with Mauchly’s test of sphericity. For the tests in which this assumption was violated, the Greenhouse–Geisser correction statistic was reported. For a parameter revealing a main effect of a condition, we calculated the mean among eight directions and conducted a paired *t*-test with the Bonferroni correction to directly compare between conditions. All statistical analyses were performed using SPSS version 26 (IBM, Armonk, NY, USA). Effects were considered significant if *p* < 0.05. Effect sizes are reported as Cohen’s *d* for paired *t*-tests and partial eta-squared value (*η*_p_^2^) for ANOVA_RM_.

## Results

**Fig 2:**
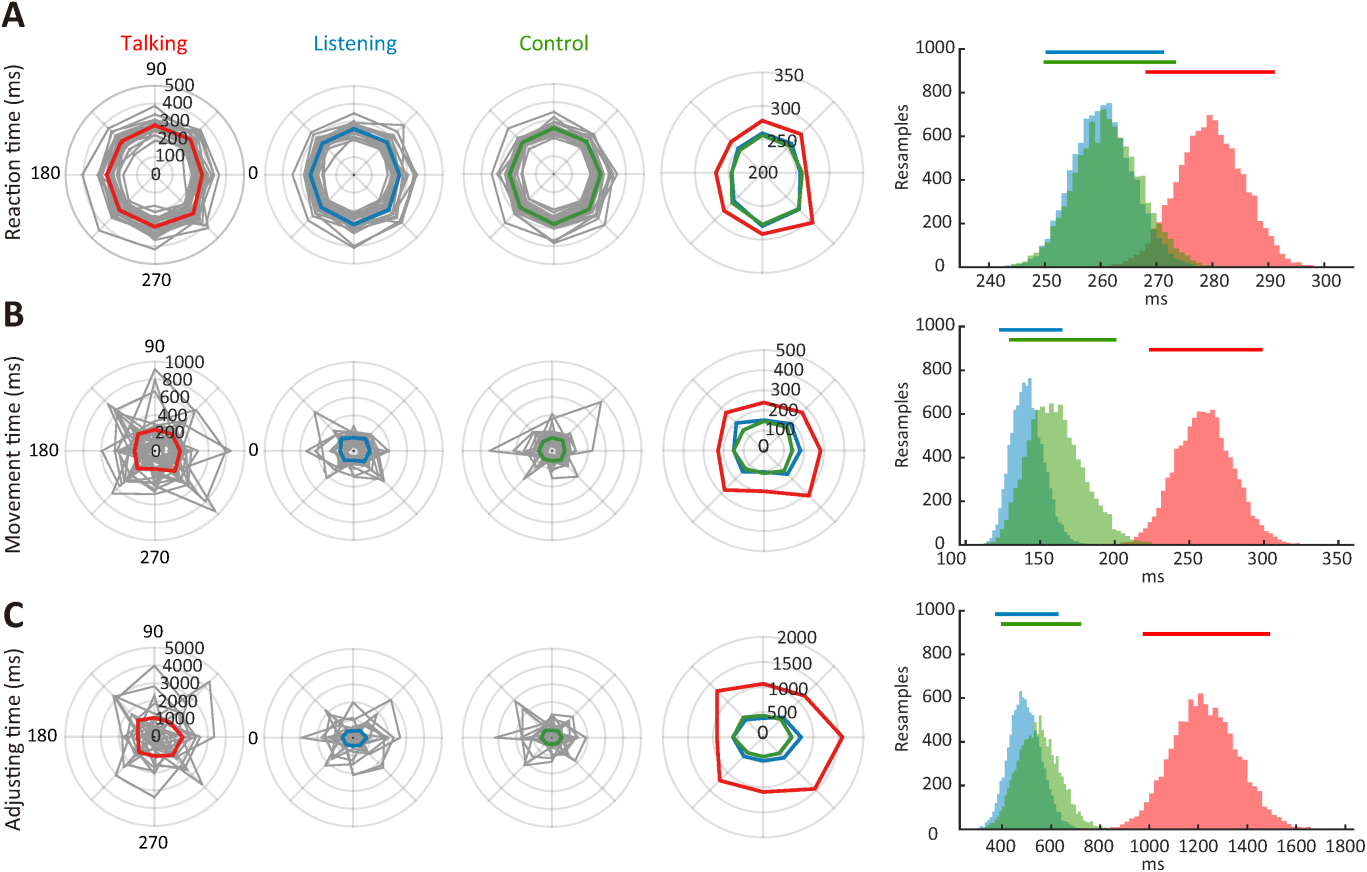
Movement parameters (A: Reaction time, B: Movement time, and C: Adjusting time) acquired during the eye movement task in three different experimental conditions. The gray lines represent the data for individual participants. The red, blue, and green lines indicate the average among participants for the talking, listening, and control conditions, respectively. The rightmost histograms are estimates of data distribution. Estimates are generated from bootstrapped 10,000 resamples of the data for each condition for display purposes. 95% confidence intervals are presented above the distributions.

When comparing the three experimental conditions, we found a longer reaction time in the talking condition than in the other conditions, irrespective of target locations (Fig 2A). In line with this, ANOVA_RM_ revealed a main effect of the condition (*F*_1.5, 42.7_ = 18.7, *p* < 0.01, *η*_p_^2^ = 0.39) but no interaction between the condition and direction (*F*_6.9, 199.5_ = 1.1, *p* = 0.32, *η*_p_^2^ = 0.04). ANOVA_RM_ also showed a main effect of direction (*F*_3.3, 95.2 _= 10.1, *p* < 0.01 *η*_p_^2^ = 0.26), demonstrating that a direction-specific delayed reaction was commonly found among conditions such as the downward directions of 225, 270, and 315 °. Post-hoc comparisons between conditions revealed a trend of a longer reaction time in the talking condition (mean 279.7 ms, SD 32.8) than in the listening (mean 260.4 ms, SD 29.7, *p* = 0.07, *d* = 0.62) and control conditions (mean 261.3 ms, SD 32.8, *p* = 0.09, *d* = 0.56); there was no significant difference between the listening and control conditions (*p* = 1.00, *d* = 0.03).

Similarly, we found a prolonged movement time when reaching in the talking condition than in the other conditions (Fig 2B). ANOVA_RM_ revealed a main effect of condition (*F*_1.4, 39.8_ = 31.5, *p* < 0.05, *η*_p_^2^ = 0.52) but no effect of direction (*F*_3.1, 90.4_ = 1.9, *p* = 0.13, *η*_p_^2^ = 0.63) or interaction between the factors (*F*_6.7, 193.5_ = 1.2, *p* = 0.29, *η*_p_^2^ = 0.41). Post-hoc comparisons between conditions revealed a longer movement time in the talking condition (mean 260.1 ms, SD 107.6) than in the listening (141.5 ms, SD 58.9, *p* < 0.05, *d* = 1.37) and control conditions (mean 160.8 ms, SD 102.1, *p* < 0.05, *d* = 0.95). There was no significant difference between the listening and control conditions (*p* = 1.00, *d* = 0.23).

Similar to the previous two parameters, the time for gaze adjustment was longer in the talking condition than in the other conditions (Fig 2C). ANOVA_RM_ showed a significant main effect of condition (*F*_1.1, 33.1_ = 35.9, *p* < 0.05, *η*_p_^2^ = 0.55) but no effect of either direction (F_3.7, 108.2_ = 1.6, *p* = 0.18, *η*_p_^2^ = 0.53) or interaction between the factors (F_4.4, 129.0_ = 0.8, *p* = 0.53, *η*_p_^2^ = 0.28). A post-hoc direct comparison revealed a longer time for gaze adjustment in the talking (mean 1226.5 ms, SD 723.3) than in the listening (mean 493.2 ms, SD 361.5, *p* < 0.05, *d* = 1.28) and control conditions (mean 548.9, SD 461.2, *p* < 0.05, *d* = 1.12). There was no significant difference between the listening and control conditions (*p* = 1.00, *d* = 0.13).

In summary, all three temporal parameters (reaction, movement, and adjusting times) of the center-out eye movements tended to be longer specifically in a situation in which participants were required to converse concurrently while performing the task than when participants were listening to audio clips or were simply performing the task. These results indicate that talking-associated cognitive loads interfered with gaze behavior in such a way that the time to initiate toward, move to, and stay on a peripheral visual target became longer.

## Discussion

The present study sought to investigate the potential effects of talking-associated cognitive loads on the temporal parameters of gaze control when responding to peripheral visual targets. The results showed that talking-associated cognitive loads delayed the time for initiating eye movements in response to a peripheral visual target and the following time for moving to and staying on the target.

Visual information has been reported to account for 90% of the information required to drive a vehicle [16], underscoring the importance of the high-quality gaze behavior—characterized by rapid and accurate control— for effectively capturing external visual information. Previous works have shown that secondary tasks involving cognitive loads influence visual functions while driving. Recarte and Nunes [24] demonstrated that verbal and spatial-imagery tasks limit the range of the functional visual field and lead drivers’ to fixate for longer. Furthermore, Harbluk et al. [18] found that cognitive loads led drivers to spend more time looking centrally ahead and less time looking to the areas in the periphery, therefore affecting vehicle control. Having a conversation while driving has also been shown to involve considerable cognitive loads that influence visual attention [25], to increase the failure to detect visual events, and/or to slow reactions to these events [20]. Moreover, cognitive load has been reported to degrade the accuracy of gaze landing [19]. Although these findings led to the hypothesis that talking-associated cognitive loads would influence gaze behavior, a specific question as to how the latency to initiate and the time to complete eye movements toward peripheral visual targets would be affected remained unaddressed. To elucidate upon this question, here, we divided the time periods related to the control of gaze behavior into reaction, movement, and adjusting times and demonstrated that all time periods became longer, particularly under the influence of talking-associated cognitive loads.

These findings support our hypothesis and suggest that talking-associated cognitive loads affect not only the initiation of the eye movements, as shown in previous work [9], but also the accuracy of the subsequent goal-directed eye movements. Though speculative, such degradation of gaze behavior may result from competition for shared attentional resources within the fronto-parietal network [26] and from reduced excitatory drive from the frontal and supplementary eye fields to the superior colliculus [27], both of which can delay the initiation of gaze shift and degrade its accuracy.

In both movement and adjusting phases, the degraded accuracy of gaze behavior under the influence of talking-associated cognitive loads was confirmed to be similar for the eight different directions. In contrast, direction-specific delays in reaction time alone were found particularly toward downward directions (i.e., 225, 270, and 315 °). This result is consistent with the classically demonstrated finding of a pronounced saccade bias in favor of the upper fields: a shorter saccade latency to upper-field targets and a greater likelihood of making an upward initial saccade [28–32]. This saccadic directional bias may lead to a global delay in the response to lower-field targets regardless of conditions. Since objects appearing on the road, such as debris or children, require a downward gaze particularly in this direction, difficulty in orienting gaze in this direction could present a critical disadvantage, particularly in driving scenarios.

The present study demonstrated no significant differences in all the three temporal parameters between the listening and control conditions. This may be attributed to the possibility that the participants did not pay considerable attention to recitals under the listening condition. In fact, it has been shown that attentional disengagement, the ability to withdraw focus from an object and is a crucial aspect of attentional control, is delayed in active listening situations in which listeners are asked to answer trivia questions [33]. Furthermore, even involuntary eye movements directing towards an object suddenly appearing during scene viewing (i.e., oculomotor capture) can be modulated by top-down attention mechanisms [34–36]. A more demanding situation where participants were forced to answer a quiz on or to tell the content after listening may require more attention and therefore result in greater interference to the present temporal parameters of gaze behavior.

The present results suggest that well-known talking-associated performance decrements while driving may in part be attributed to delayed reaction times and degraded accuracy of subsequent gaze behavior in the visuomotor processes required for driving. Considering the role of gaze behavior in driving situations, we assume that talking-associated deterioration in gaze behavior seems to indirectly influence driving maneuvers such as physical responses (e.g., stepping on the brake, turning the steering wheel, etc.) in response to visual events. It is plausible that the delayed response and subsequent degraded gaze behavior may lead to a prolonged time before the recognition of visual objects, such as the contents of billboards or things on the side of the road, which would likely lead to the delayed initiation of physical responses. Nevertheless, it is also possible that talking may slow down driving-related motor responses independently of any changes in the latency and accuracy of gaze behavior. For instance, a braking reaction would be delayed while talking even under a situation where a driver is staring at the taillights on a lead car, due to hindered visual encoding by the distraction of attention (i.e., inattentional blindness, [25,37]). Therefore, it should be in mind that the interference of gaze behavior may not be the sole or primary cause of slowed physical responses of driving, but rather more common mechanisms may attribute to them [38,39].

Based on the present findings, talking situations undoubtedly involve greater cognitive loads than simple listening situations. However, the extent of the load in each individual could not be quantified, as the study did not record the number of questions posed to each participant or the number they were unable to answer; Consequently, the present study cannot clarify the threshold or the extent of the cognitive load that initiates interference with reactions and following gaze behavior. Additionally, the question of whether the effect is primarily due to the talking behavior itself or the cognitive load associated with talking remains unresolved. Several physiological proxies have been used to estimate cognitive status, such as event-related potentials, optical imaging, heart rate, heart rate variability, blood pressure, skin conductance, electromyography, and thermal imaging [40]. In addition, eye tracking measures (e.g., fixation duration, saccade velocity, pupil dilation, and blink rate) can also be studied to detect participants’ cognitive loads [41,42]. Involuntary eye movements such as the blink rate and pupil dilation are likely to be used as a proxy for the cognitive load even during a task that requires voluntary eye movements such as that used in the present study. Quantifying the talking-associated cognitive loads and their threshold of interference is an interesting topic of research to be addressed in future studies. Another interesting point would be whether, and if so, to what extent the cognitive operations influence on gaze behavior. Indeed, a previous study demonstrated that the amount of interference to driving behavior changes depending on types of talking contents as well as the extent of cognitive loads [43].

## Conclusions

The present study demonstrated delays in the temporal parameters of gaze behavior in a demanding situation in which a fast and accurate gaze and verbal communication were concurrently required. These findings suggest that talking-associated cognitive loads have an impact on neural processes relevant in initiating and controlling gaze behavior, which are the first steps in visuomotor processing while driving.

## Supporting information

S1 Datasets The datasets generated and analyzed in the present study. (XLSX)
